# Publisher Correction: Liquid phase blending of metal-organic frameworks

**DOI:** 10.1038/s41467-018-07000-8

**Published:** 2018-10-18

**Authors:** Louis Longley, Sean M. Collins, Chao Zhou, Glen J. Smales, Sarah E. Norman, Nick J. Brownbill, Christopher W. Ashling, Philip A. Chater, Robert Tovey, Carola-Bibiane Schönlieb, Thomas F. Headen, Nicholas J. Terrill, Yuanzheng Yue, Andrew J. Smith, Frédéric Blanc, David A. Keen, Paul A. Midgley, Thomas D. Bennett

**Affiliations:** 1 Department of Materials Science and Metallurgy, University of Cambridge, Charles Babbage Road, Cambridge, CB3 0FS UK; 2 Department of Chemistry and Bioscience, Aalborg University, DK-9220 Aalborg, Denmark; 3 Department of Chemistry, University College London, Gordon Street, London, WC1H 0AJ UK; 4 Diamond Light Source Ltd, Diamond House, Harwell Science and Innovation Campus, Didcot, OX11 0DE UK; 5 ISIS Facility, Rutherford Appleton Laboratory, Harwell Campus, Didcot, OX11 0QX UK; 6 Department of Chemistry, University of Liverpool, Crown Street, Liverpool, L69 7ZD UK; 7 Department of Applied Mathematics and Theoretical Physics, Centre for Mathematical Sciences, Wilberforce Road, Cambridge, CB3 0WA UK; 8 State Key Laboratory of Silicate Materials for Architectures, Wuhan University of Technology, 430070 Wuhan, China; 9 School of Materials Science and Engineering, Qilu University of Technology, 250353 Jinan, China; 10 Stephenson Institute for Renewable Energy, University of Liverpool, Crown Street, Liverpool, L69 7ZD UK

Correction to: *Nature Communications* 10.1038/s41467-018-04553-6; published online 15 June 2018

The original version of this Article contained an error in Fig. 1b, where the blue ‘(ZIF-4-Zn)_0.5_ (ZIF-62)_0.5_ blend’ data curve was omitted from the enthalpy response plot. This has now been corrected in both the PDF and HTML versions of the Article.

