# Supplementary material for: Liquid phase blending of metal-organic frameworks
Source: Nat Commun. 2018 Jun 15;9:2135. doi: 10.1038/s41467-018-04553-6 (PMC6004012; doi:10.1038/s41467-018-04553-6)
Supplement: Supplementary file 1 — Supplementary Information [file 41467_2018_4553_MOESM1_ESM.pdf]

Supplementary Information

Liquid Phase Blending of Metal-Organic Frameworks

Louis Longley et al,

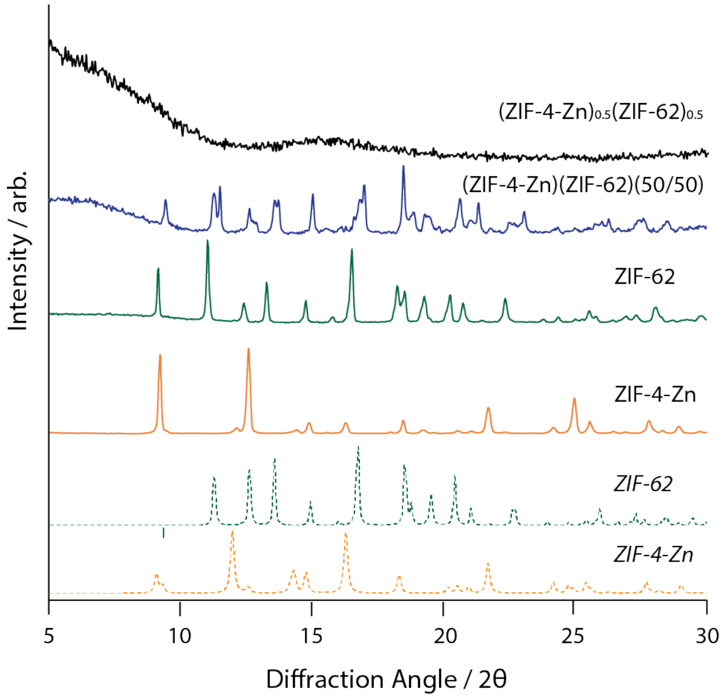

Supplementary Figure 1. X-ray powder diffraction patterns of crystalline and blended samples, along with the simulated structures (solid and broken traces respectively). The tick mark on the simulated trace of ZIF-62 indicates the presence of a Bragg peak of low intensity.

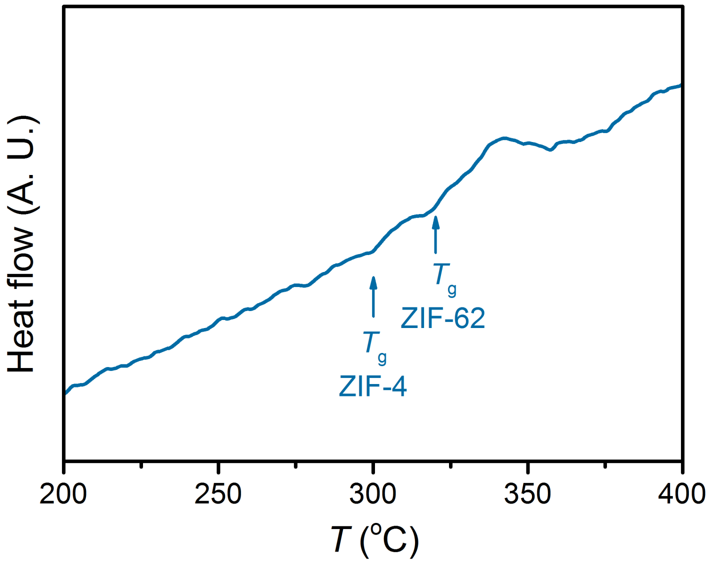

Supplementary Figure 2. DSC trace of a physical mixture formed by mixing equal weights of ZIF-62 and ZIF-4 glasses (pre-formed).

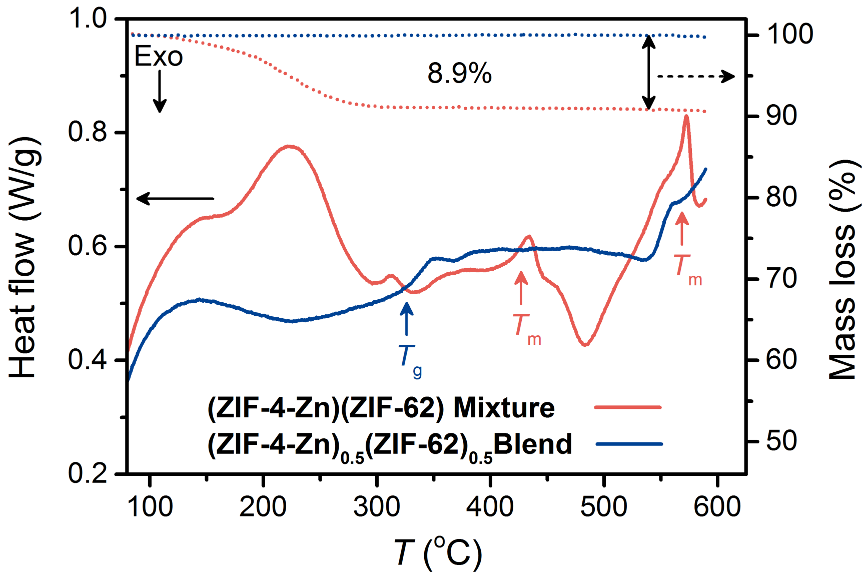

Supplementary Figure 3. Enthalpy response (red curve) and mass change (dotted curve) in the physical mixture (ZIF-4-Zn)(ZIF-62)(50/50) during heating at 10 °C min^{-1}. Blue curve: reheating curve representing the enthalpy response of the corresponding glass that forms upon quenching, i.e. (ZIF-4-Zn)$_{0.5}$(ZIF-62)$_{0.5}$ during prior cooling at 10 °C min^{-1}. This is the same data as presented in Fig. 1b, but with a higher magnification for comparison with Supplementary Figure 3.

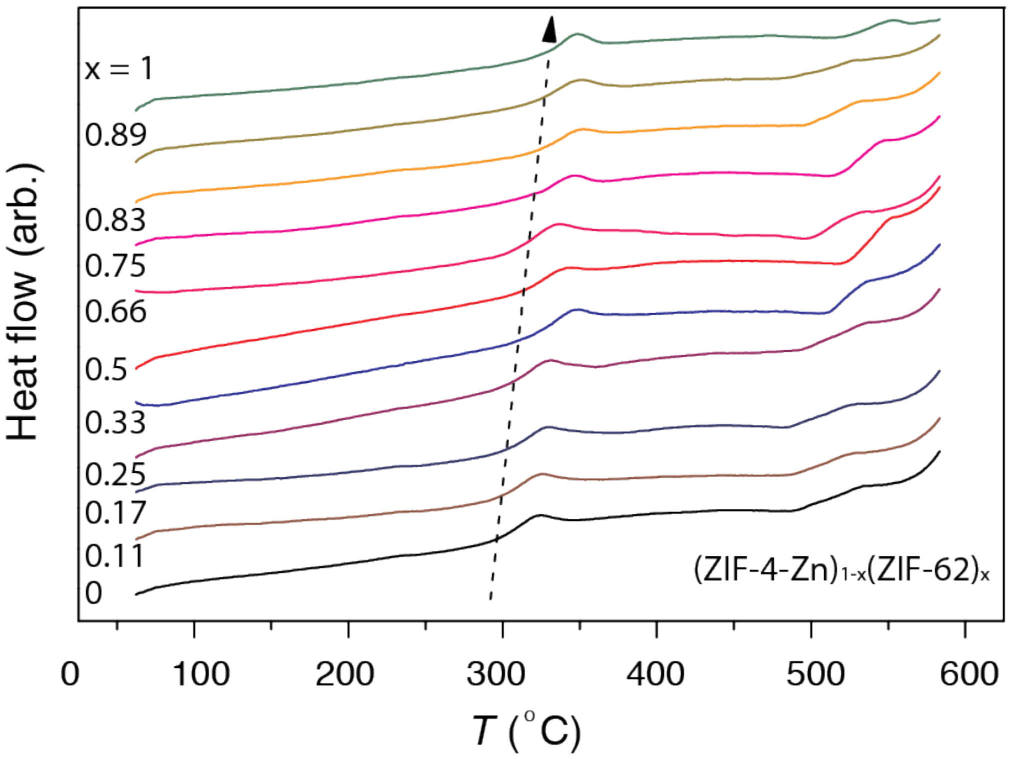

Supplementary Figure 4. DSC upscans of glasses of the sample series $(ZIF\text{-}4\text{-}Zn)_{1-x}(ZIF\text{-}62)_x$. Heating rate 10 °C min^{-1}.

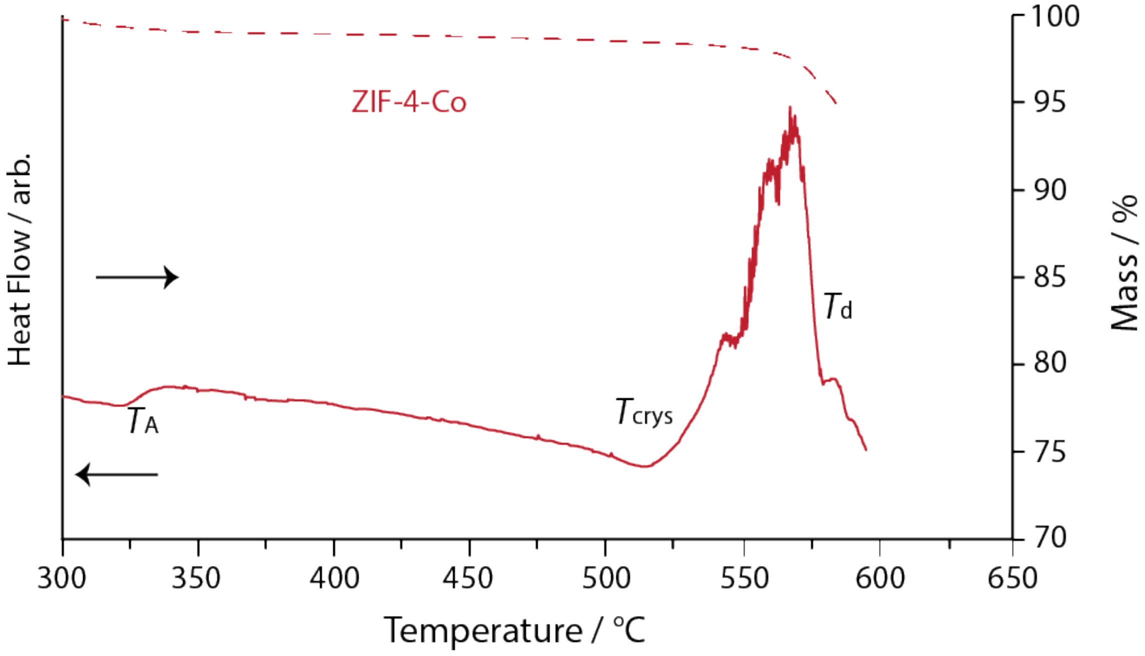

Supplementary Figure 5. Simultaneous DSC/TGA of ZIF-4-Co. Heating rate 10 °C min^{-1}.

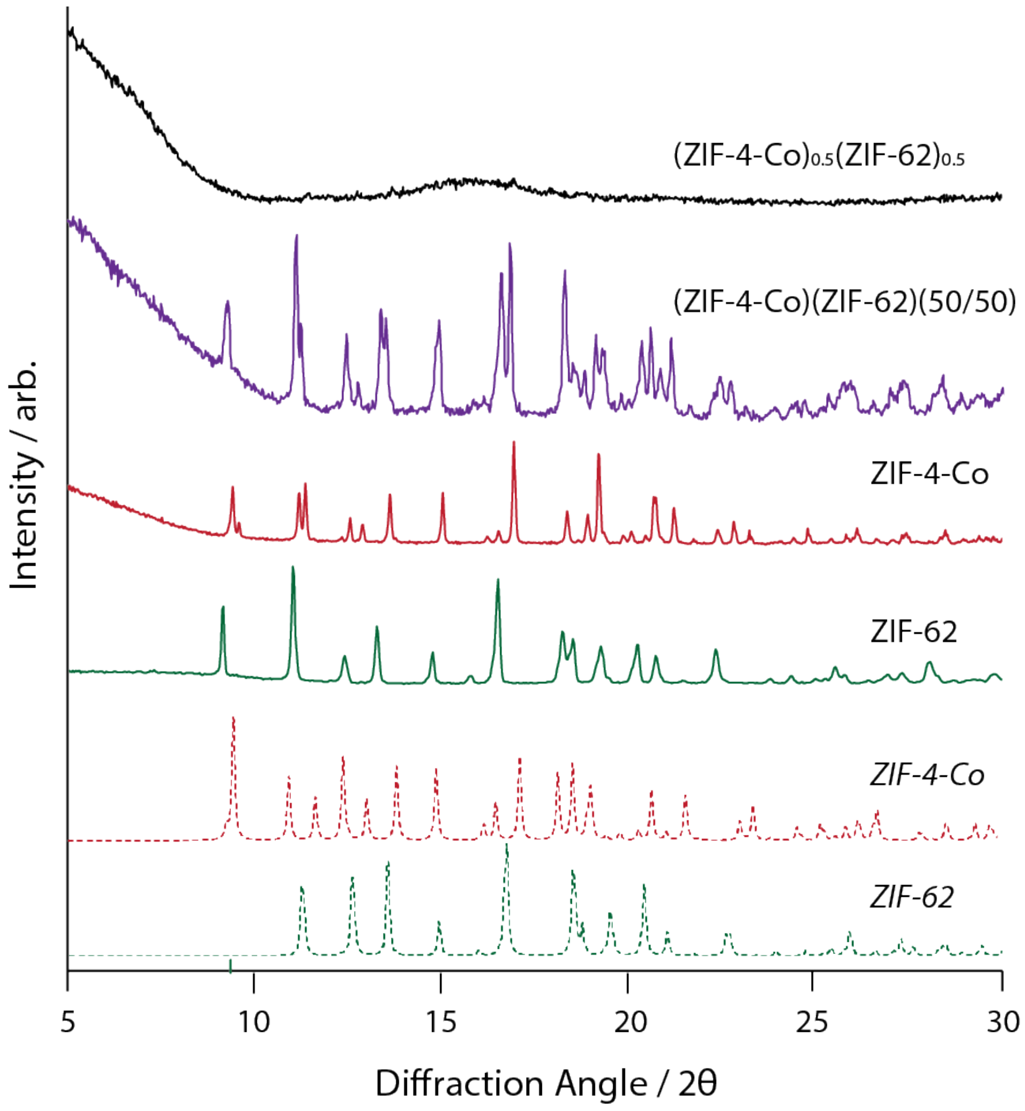

Supplementary Figure 6. X-ray powder diffraction patterns of ZIF-62 (green), ZIF-4-Co (red), (ZIF-4-Co)(ZIF-62)(50/50) (purple) and $(ZIF\text{-}4\text{-}Co)_{0.5}(ZIF\text{-}62)_{0.5}$ (black). Extremely small Bragg peaks can be seen in the diffraction pattern, though the impact of them cannot be seen in the PDF.

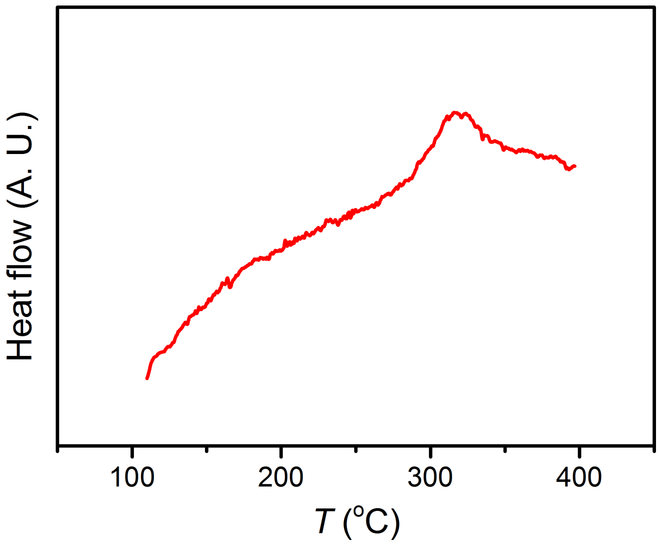

Supplementary Figure 7.. DSC trace of $(ZIF\text{-}4\text{-}Co)_{0.5}(ZIF\text{-}62)_{0.5}$, conducted at a heating rate 10 °C min^{-1} heating rate.

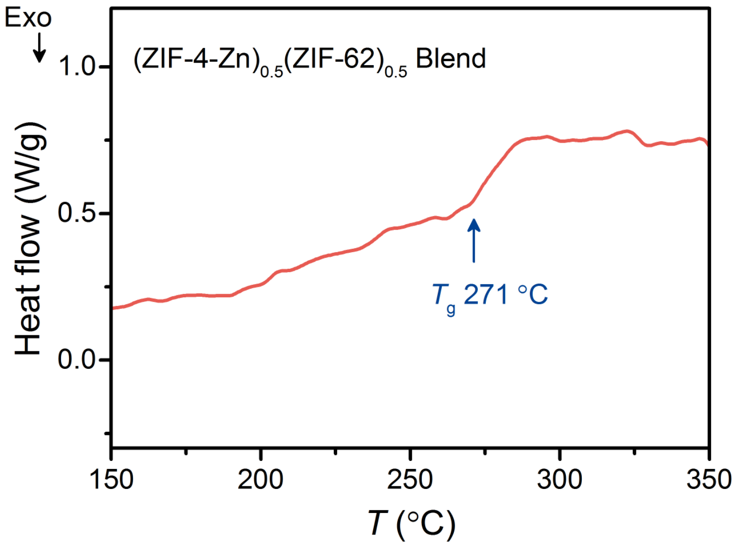

Supplementary Figure 8. DSC trace of $(ZIF\text{-}4\text{-}Co)_{0.5}(ZIF\text{-}62)_{0.5}$, conducted at 5 °C min^{-1} heating rate.

a

b

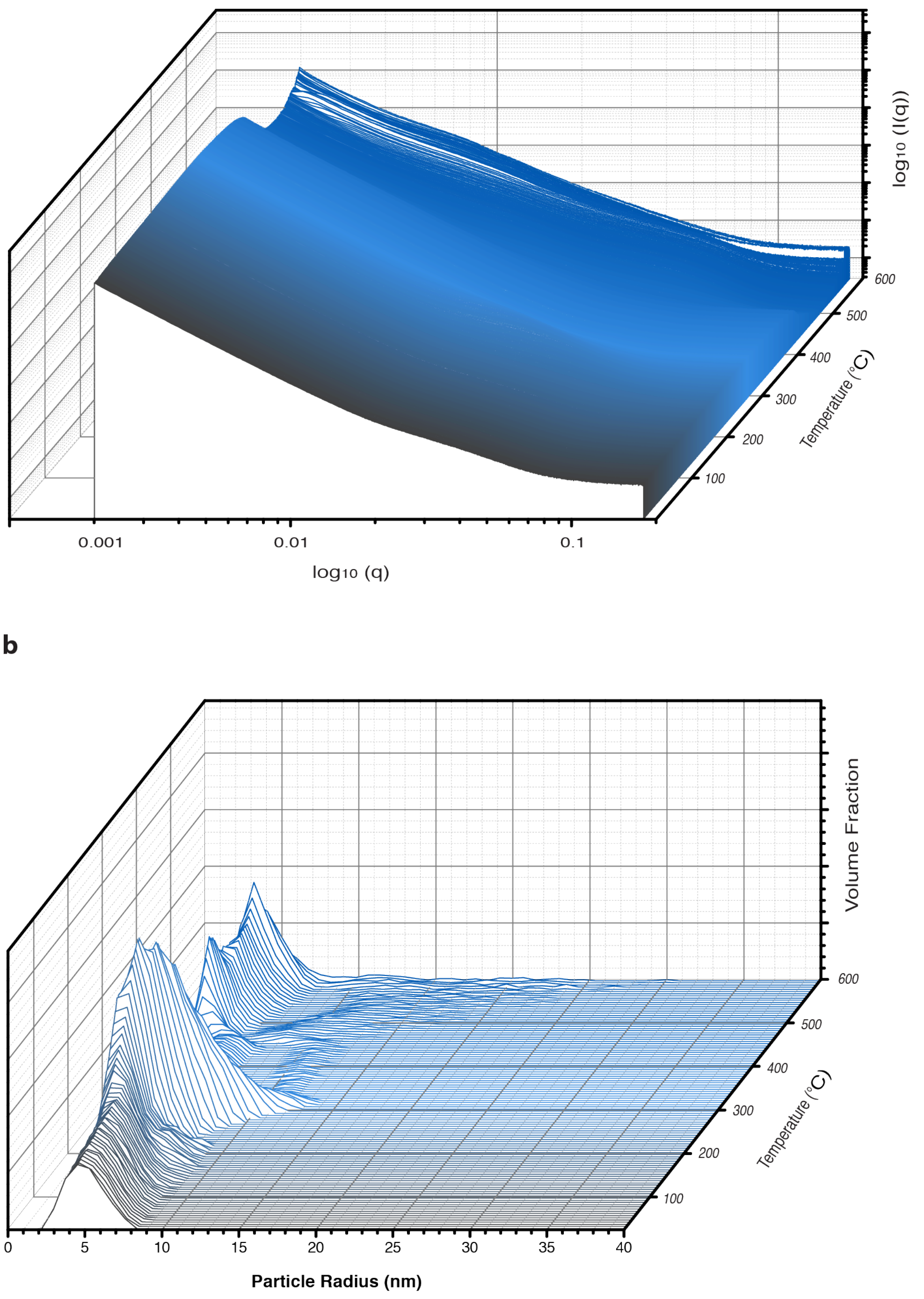

Supplementary Figure 9. Temperature resolved (a) SAXS profile and (b) volume fraction distributions of ZIF-62.

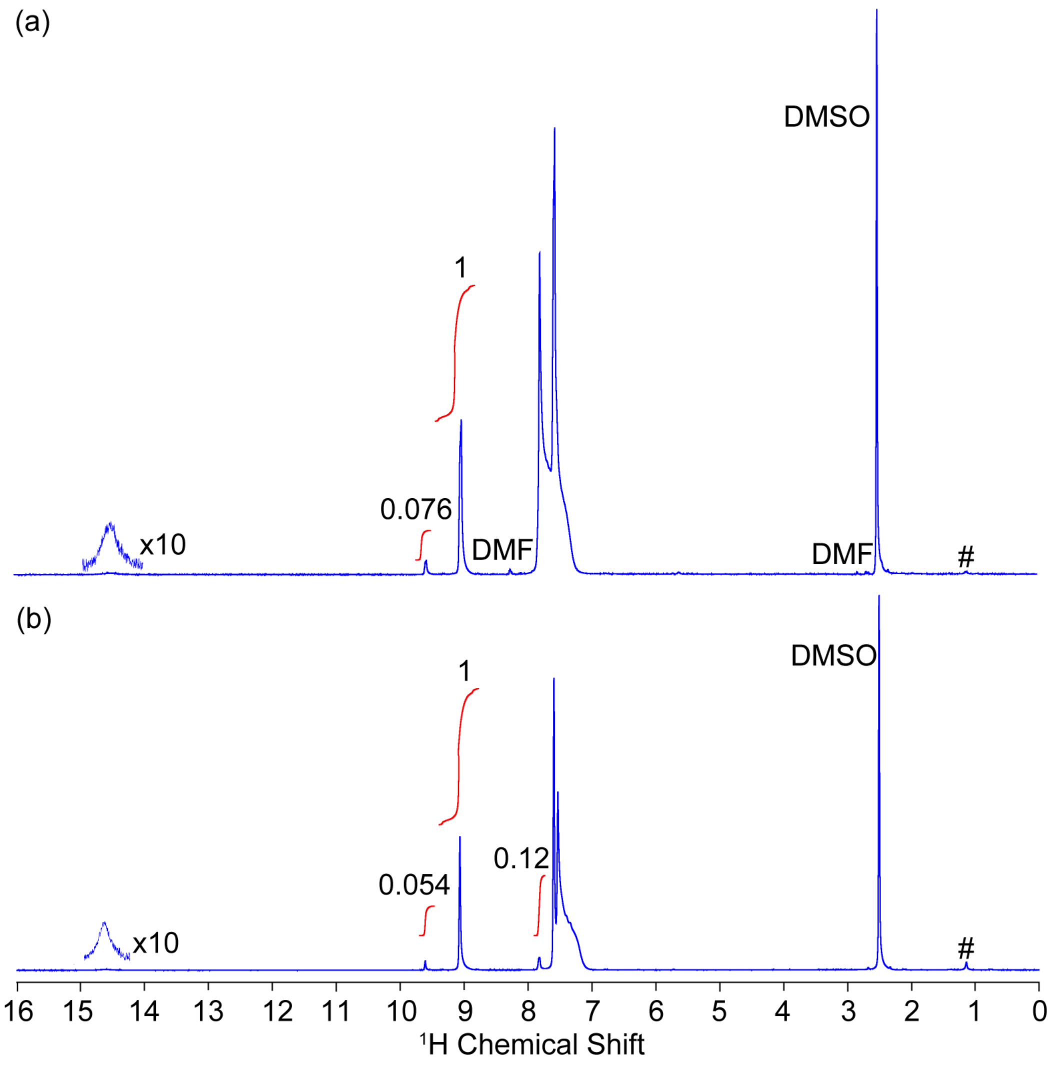

Supplementary Figure 10. Liquid-state ^{1}H NMR spectra of (a) (ZIF-4-Co)(ZIF-62)(50/50) and (b) (ZIF-4-Co)$_{0.5}$(ZIF-62)$_{0.5}$, (see below for complete spectral assignment). Integrations of individual ^{1}H shown in red only when resolution allowed. Solvent peaks are labelled, and # denote resonances from small amount of grease.

(ZIF-4-Co)(ZIF-62)(50/50): ^{1}H NMR (400 MHz, DMSO-d_6): δ (ppm) 1.14 (grease), 2.51 (DMSO), 2.63, 2.81 (DMF), 7.39 (C***H***CHCN$_{\text{bIm}}$), 7.52 (HCl/H_2O), 7.57 (C***H***C***H***N$_{\text{Im}}$), 7.78 (C***H***CN$_{\text{bIm}}$), 8.23 (DMF), 9.02 (NC***H***N$_{\text{Im}}$), 9.57 (NC***H***N$_{\text{bIm}}$), 14.49 (N***H***$_{\text{Im/bIm}}$).

(ZIF-4-Co)$_{0.5}$(ZIF-62)$_{0.5}$: ^{1}H NMR (400 MHz, DMSO-d_6): δ (ppm) 1.14 (grease), 2.51 (DMSO), 7.39 (C***H***CHCN$_{\text{bIm}}$), 7.52 (HCl/H_2O), 7.59 (C***H***C***H***N$_{\text{Im}}$), 7.82 (C***H***CN$_{\text{bIm}}$), 9.07 (NC***H***N$_{\text{Im}}$), 9.61 (NC***H***N$_{\text{bIm}}$), 14.59 (N***H***$_{\text{Im/bIm}}$).

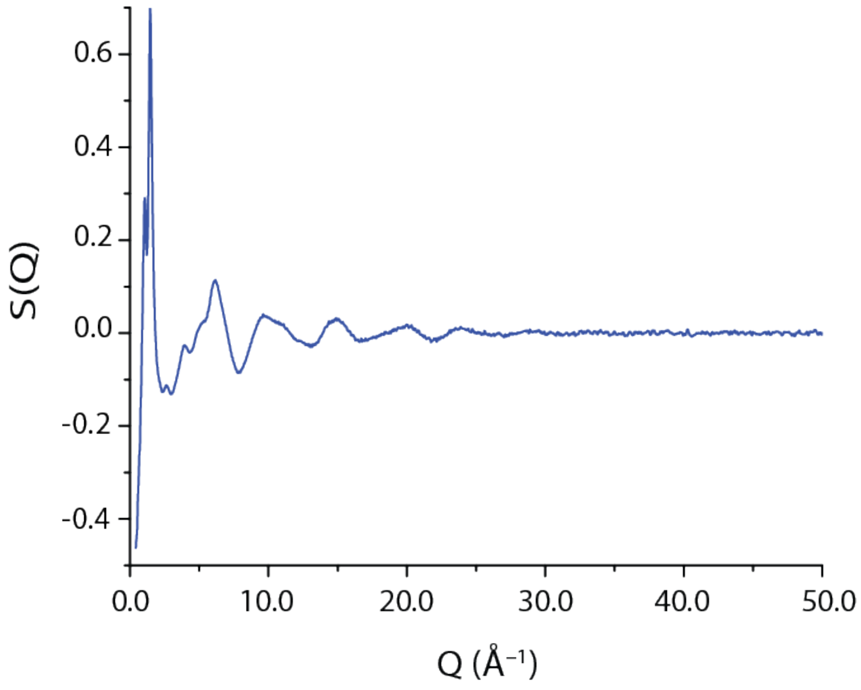

Supplementary Figure 11. Neutron total scattering structure factor *S*(*Q*) of (ZIF-4-Co)$_{0.5}$(ZIF-62)$_{0.5}$.

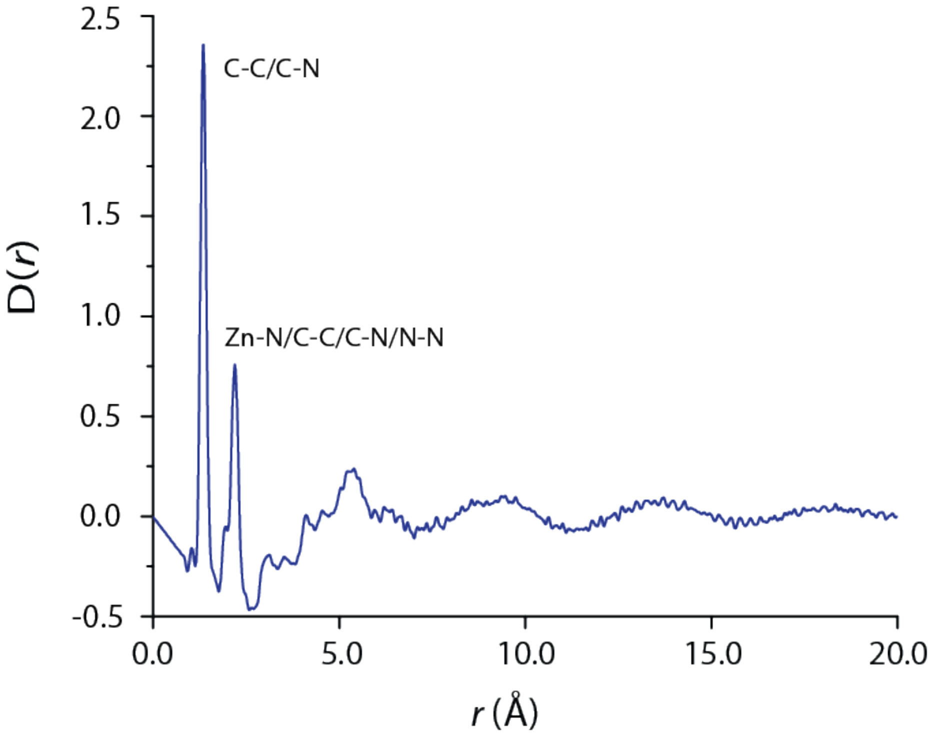

Supplementary Figure 12. Neutron pair distribution function *D*(*r*) of (ZIF-4-Co)$_{0.5}$(ZIF-62)$_{0.5}$.

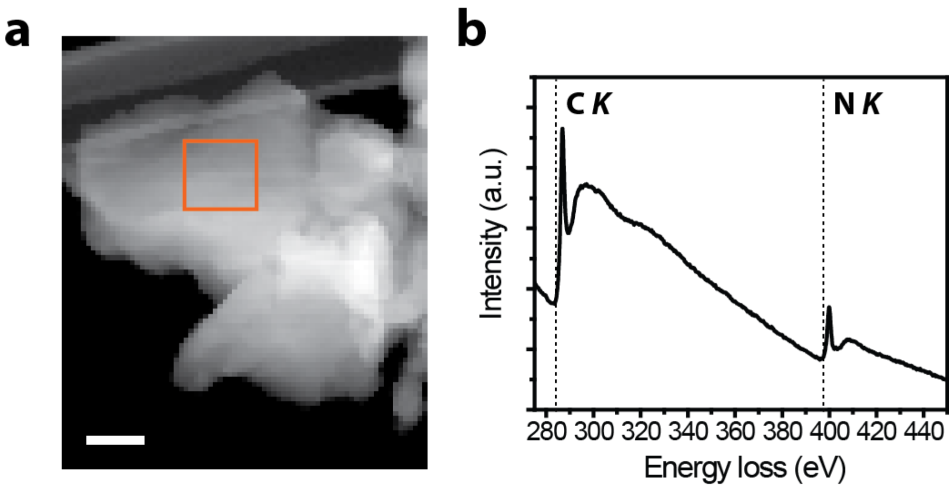

Supplementary Figure 13. **Electron Microscopy on Pure ZIF-62 Crystal and Glass Samples**. (a) ADF-STEM and (b) EELS characterization of a crystalline ZIF-62 particle supported on lacey carbon. The spectrum in (b) is from the boxed region in (a) with no contribution from the lacey carbon support. The scale bar is 100 nm.

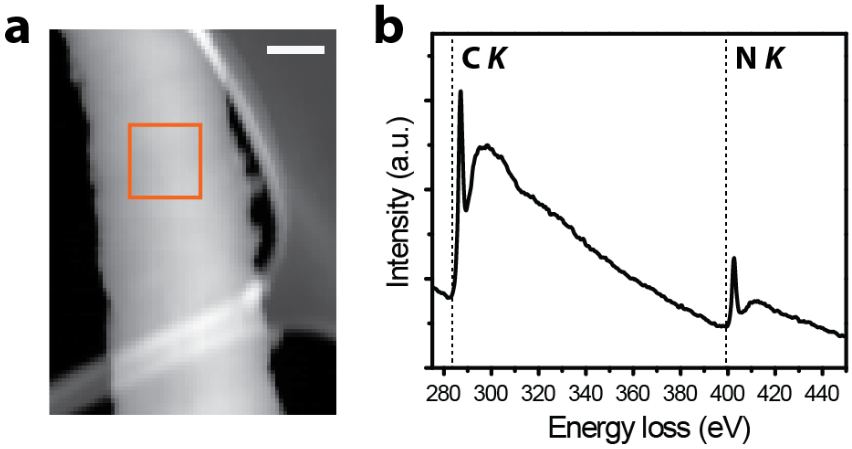

Supplementary Figure 14. (a) ADF-STEM and (b) EELS of a ZIF-62 glass particle supported on lacey carbon. The spectrum in (b) is from the boxed region in (a) with no contribution from the lacey carbon support. The scale bar is 100 nm.

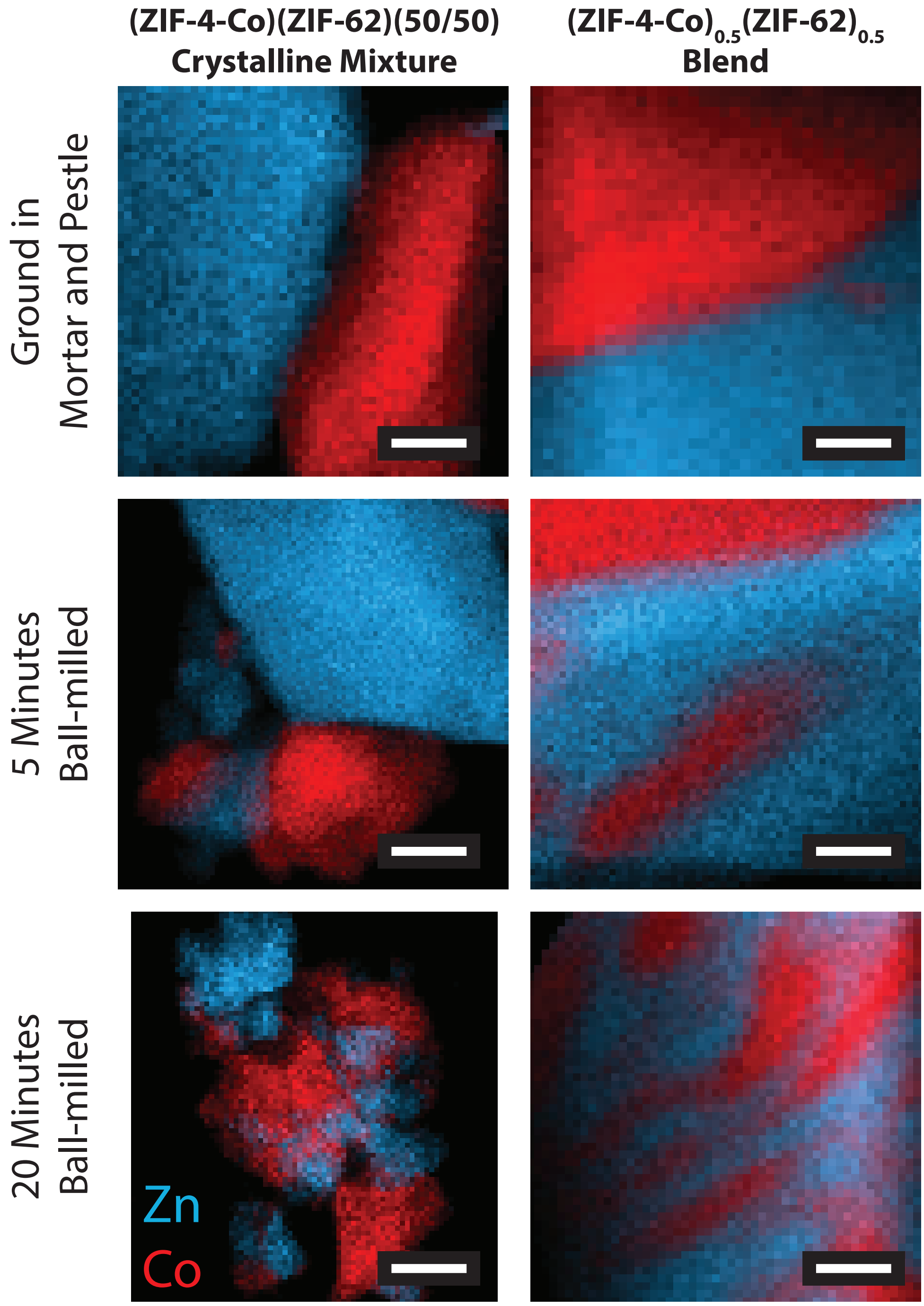

Supplementary Figure 15. ADF-STEM image of samples of (ZIF-4-Co)(ZIF-62)(50/50) formed by (top) lightly grinding crystalline ZIF-4-Co and ZIF-62 in a mortar and pestle, (middle) ball-milling together for 5 minutes and (bottom) ball-milling together for 20 minutes. Maps were generated by peak integration at the K_α X-ray lines for Co and Zn, shown as an overlay. The right hand column contains identical analysis of the glass samples. Scale bars correspond to 200 nm.

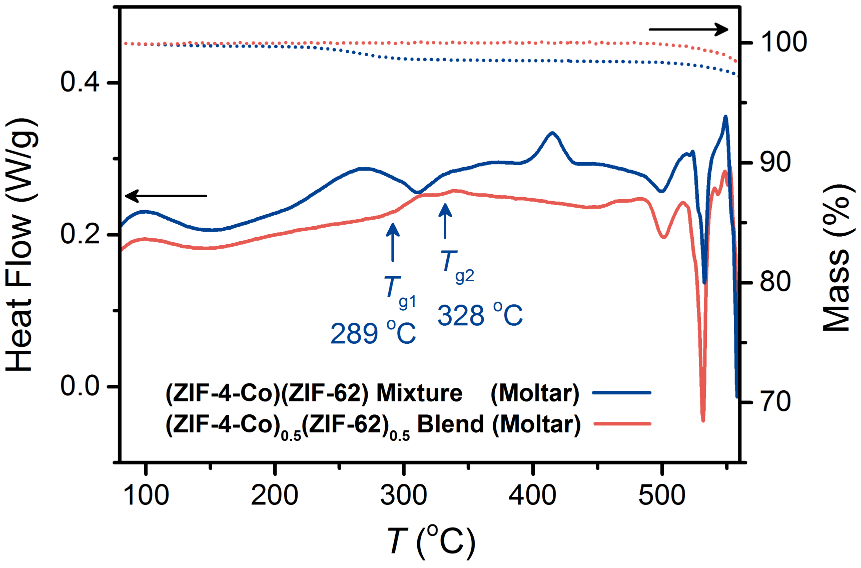

Supplementary Figure 16. DSC scan on a sample of **(**ZIF-4-Co)(ZIF-62)(50/50) formed by hand grinding, and the resultant glass, conducted at 10 °C min^{-1} heating rate. The second glass transition temperature T_{g2} is defined as the intersection point between lines constructed from the (constant) heatflow after T_{g1} and before the next increase in heat flow, and a line drawn at a tangent to the point at which the heat flow increase is ½ of the total (see Supplementary Figure 17 below).

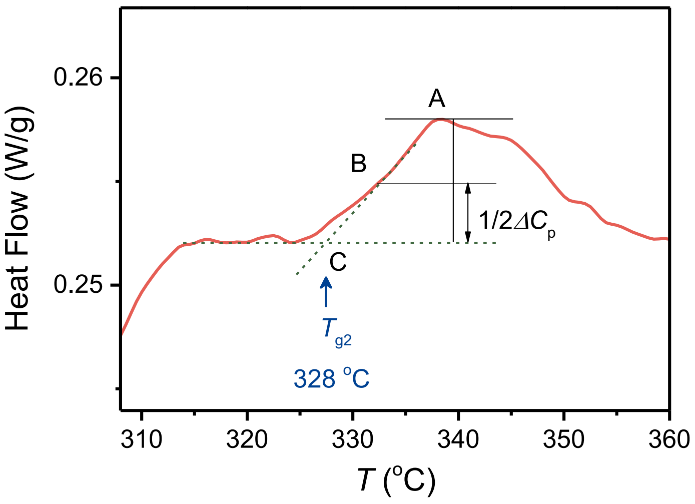

Supplementary Figure 17. Second glass transition point construction.

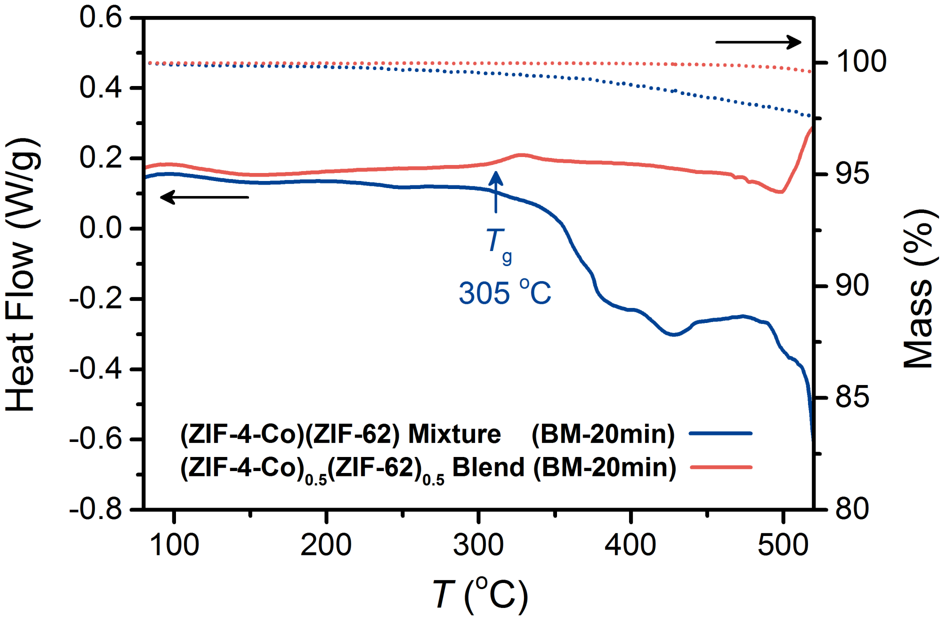

Supplementary Figure 18. DSC scan on a sample of **(**ZIF-4-Co)(ZIF-62)(50/50) formed by 20 minutes of ball-milling, and the resultant glass, conducted at 10 °C min^{-1} heating rate.
